# Supplementary material for: Aluminum corrosion–passivation regulation prolongs aqueous batteries life
Source: Nat Commun. 2024 Apr 4;15:2922. doi: 10.1038/s41467-024-47145-3 (PMC10995134; doi:10.1038/s41467-024-47145-3)
Supplement: Supplementary file 1 — Supplementary Information [file 41467_2024_47145_MOESM1_ESM.pdf]

Aluminum Corrosion-Passivation Regulation Prolongs Aqueous Batteries Life

Binghang Liu[1,2,3], Tianshi Lv[1,2,3], Anxing Zhou[1,2,3], Xiangzhen Zhu[1,2,3], Zejing Lin[1,2], Ting Lin[1], Liumin Suo[1,2,3*]

1 Beijing National Laboratory for Condensed Matter Physics, Institute of Physics, Chinese Academy of Science, Beijing 100190, China

2 Center of Materials Science and Optoelectronics Engineering, University of Chinese Academy of Sciences, Beijing 100049, China

3 Yangtze River Delta Physics Research Center Co. Ltd, Liyang 213300, China

* Email: suoliumin@iphy.ac.cn

Supplementary Note 1. Comparison of price, conductivity, and density among Al, SS, Ti, and Ni 2

Supplementary Note 2. pH and anti-corrosion properties of electrolytes with HTA 4

pH of LiTFSI solutions with HTA 4

The discussion of the role of HTA 6

The analysis of the amounts of HTA 7

Passivation layer durability validation 8

Repeatability of the CA experiments 13

The verification of the anti-corrosion effect of Na_2CO_3 and Li_2CO_3 in 1m NaTFSI 16

Supplementary Note 3. Identify and quantify the Al oxidation corrosion for lithium compensation 19

Identify the Al corrosion in the battery curves. 19

Quantify the Al corrosion for lithium compensation 21

Supplementary Note 4. Illustrate the prototype of self-prolonging aqueous Li-ion batteries and check the performance of Al current collector in the cycling 25

Validating the feasibility of the sacrificial pre-lithiation Al electrode in the battery 25

Repeatability verification of 0.5Ah SP-ALIB 27

The over-lithiation plateau in 0.5 Ah $LiMn_2O_4$-TiO_2 pouch cell 29

The cycling stability comparison of Al + HTA with Ti current collector without over-lithiation 31

Supplementary figures

Supplementary Note 1. Comparison of price, conductivity, and density among Al, SS, Ti, and Ni

The price, conductivity and density of Al, SS, Ti, and Ni are shown in Figure S1. Al is far superior to the other three in terms of all three parameters. SS, Ti, and Ni have outstanding corrosion resistance but cannot satisfy all the requirements for the battery. Considering energy density, internal resistance, and cost of industrial battery production, Al is the most promising candidate. Solving the corrosion problem is the final step in aluminum's ascent to the top.

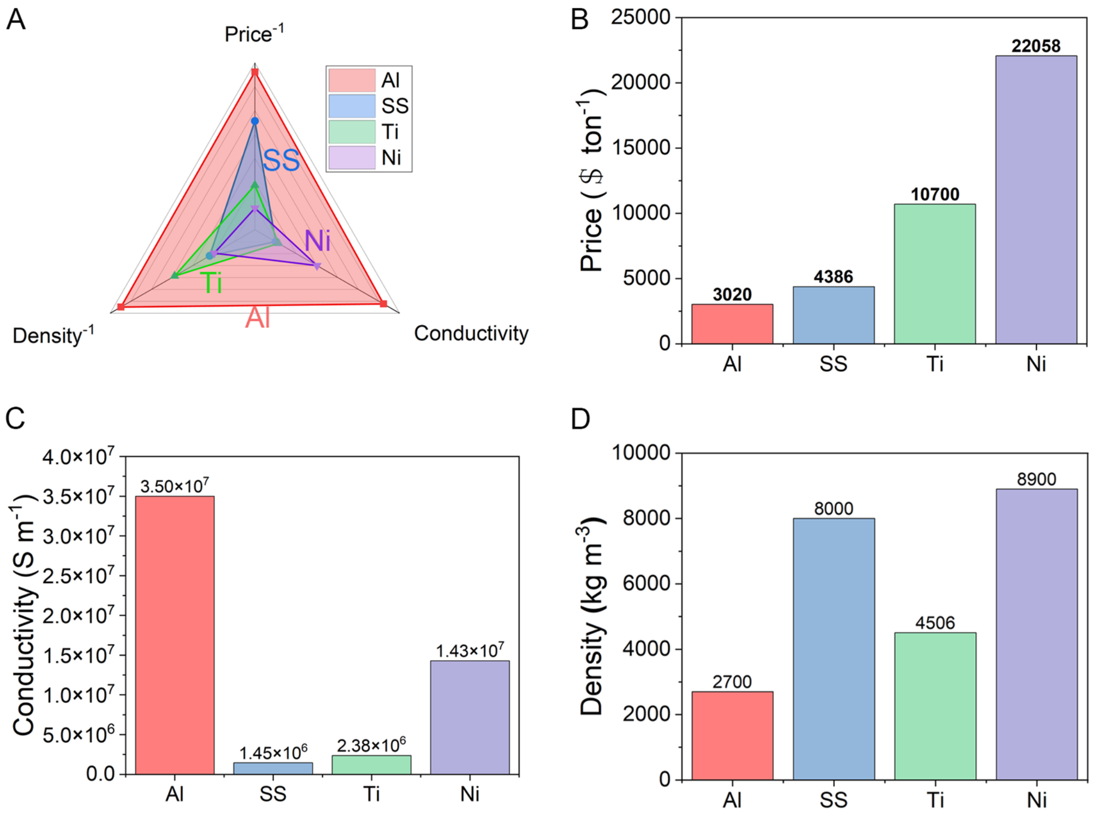

Figure S1. Comparison of price, conductivity and density among Al, SS, Ti, and Ni. (A) Comparison of price^{-1}, density^{-1} and conductivity among Al, SS, Ti, and Ni. (B) Comparison of price. (C) Comparison of conductivity. (D) Comparison of density. 304 stainless steel is chosen to present SS. The Price of Al, SS and Ni is selected from August 2022 data[1-3] while the price of Ti is from the second quarter of 2022[4].

Supplementary Note 2. pH and anti-corrosion properties of electrolytes with HTA

pH of LiTFSI solutions with HTA

The pH of LiTFSI solutions with HTAs at 25℃ is shown in Figure S2. In low-concentration solutions like 1m, HTA increases pH. In 10m LiTFSI, it's hard for additives to adjust pH because the higher the concentration of Li^+, the harder it is to dissolve lithium-containing additives. Free water is also less in higher concentration electrolytes, which is another reason why pH is harder to adjust in 10m LiTFSI than in 1m.

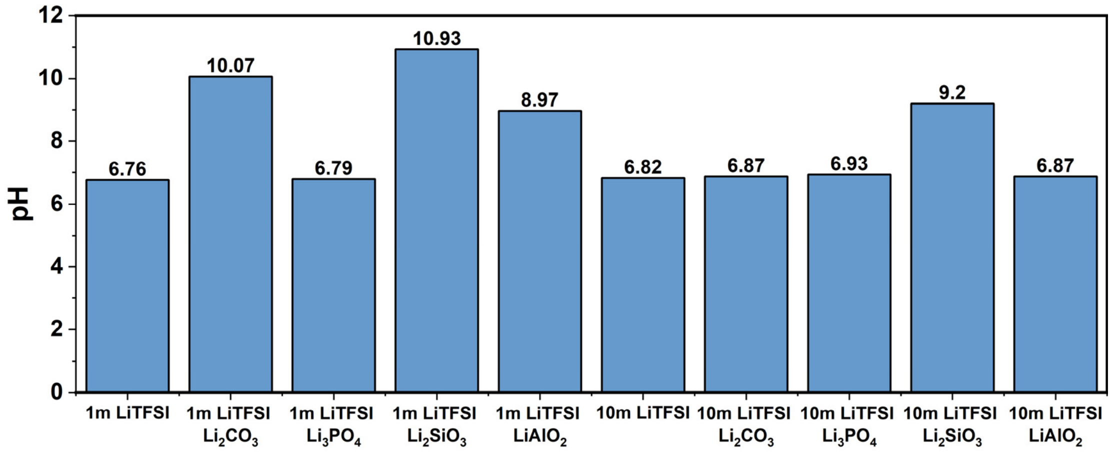

Figure S2. pH of LiTFSI solutions with saturated additives.

The discussion of the role of HTA

HTA is a catalyst for the hydrolysis of Al^{3+} and a pH buffer near Al. The reason is rooted in experimental results: $Al(OH)_3$ is not observed on Al foil after corrosion in 10m LiTFSI but observed after adding Li_2CO_3 (Figure 2E). This is not due to a significant pH difference of electrolytes in the bulk but rather possibly arises from the catalytic action of HTA and its role in microscale pH buffering.

The pH data from Figure S2 indicate a slight increase upon the addition of HTA. This is due to the ability of HTA to bind with H^+ (as pH buffer). Therefore, when the working electrode is at a high potential, the anions of HTA aggregate near the electrode, potentially amplifying this ability and facilitating the formation of $Al(OH)_3$.

The analysis of the amounts of HTA

This section discusses whether the HTA less than 0.05m is sufficient in the electrolyte to passivate the Al current collector.

We believe that HTA less than 0.05m is sufficient for passivating Al. Take HCO_3^- for example, it tends to play a catalytic role. The charging process attracts HCO_3^- to the Al current collector at positive electrode. HCO_3^- assists in the hydrolysis of Al^{3+} to form $Al(OH)_3$, while itself transforms into H_2CO_3 and diffuses into the bulk electrolyte. Corresponding equation is: $Al^{3+}(aq) + HCO_3^-(aq) + H_2O \rightarrow Al(OH)_3 + H_2CO_3$. Then the diffused H_2CO_3 dissociate back to HCO_3^- in the bulk electrolyte to maintain the catalytic activity of HCO_3^-. Therefore, HTA should not be limited by the current quantity. Therefore, there is no need to worry about the low content of HTA additive affecting the passivation effect. It is not consumed. It mainly serves as a catalyst and pH buffer.

Passivation layer durability validation

To further check the anti-corrosion effect of the passivation layer generated by HTA, we collected the Al foils that were conducted by the CA experiments in 1m LiTFSI with HTAs, washed them in ethanol and then conducted the same CA experiments in 1m LiTFSI without additives. All the CA experiments follow the same conditions as that in Figure 2. Related data are shown in Figures S3, S4, and S5.

SEM photos of Al current collectors after battery cycling are shown in Figure S6.

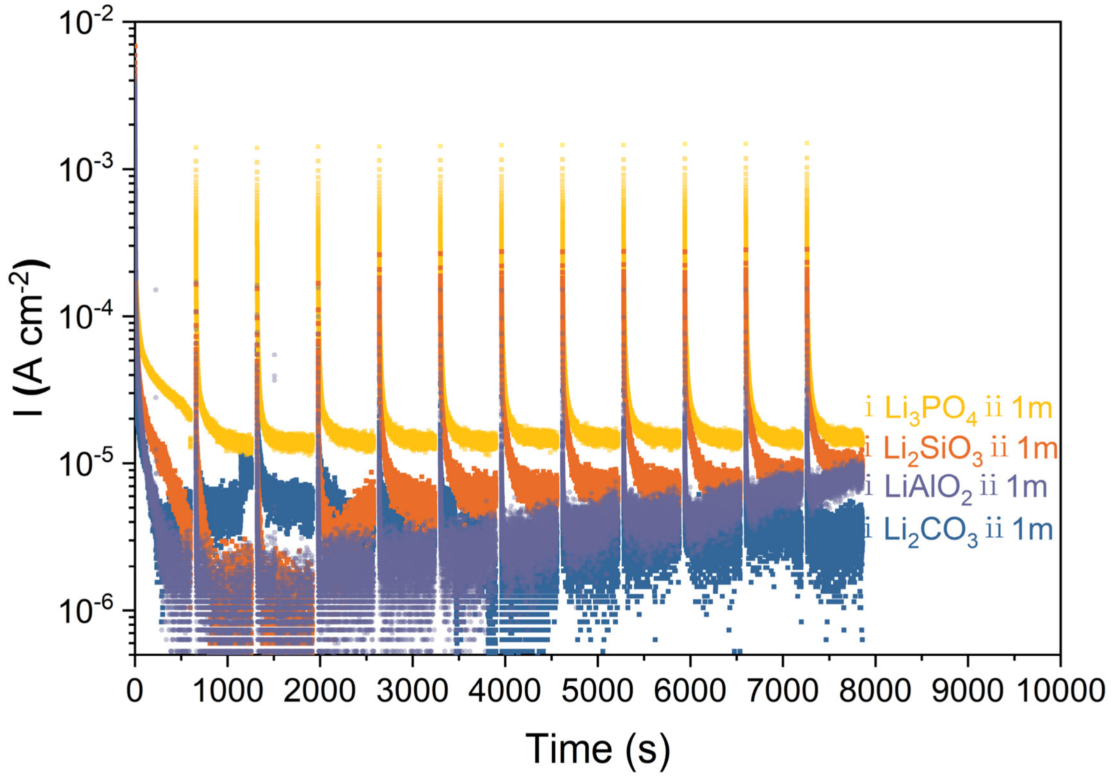

Figure S3. Chronoamperometry (CA) experiments in 1 m LiTFSI solution with Al foils that were conducted in the same CA experiments in 1m LiTFSI with saturated HTA (Li_3PO_4, Li_2CO_3, $LiAlO_2$, and Li_2SiO_3) first, and then washed in ethanol. i and ii represent the sequence of CA experiments conducted using different electrolytes. Eg: i Li_3PO_4 ii 1m means conducting the CA experiment with Al foil in 1m LiTFSI + Li_3PO_4 first, followed by conducting another CA experiment in 1m LiTFSI.

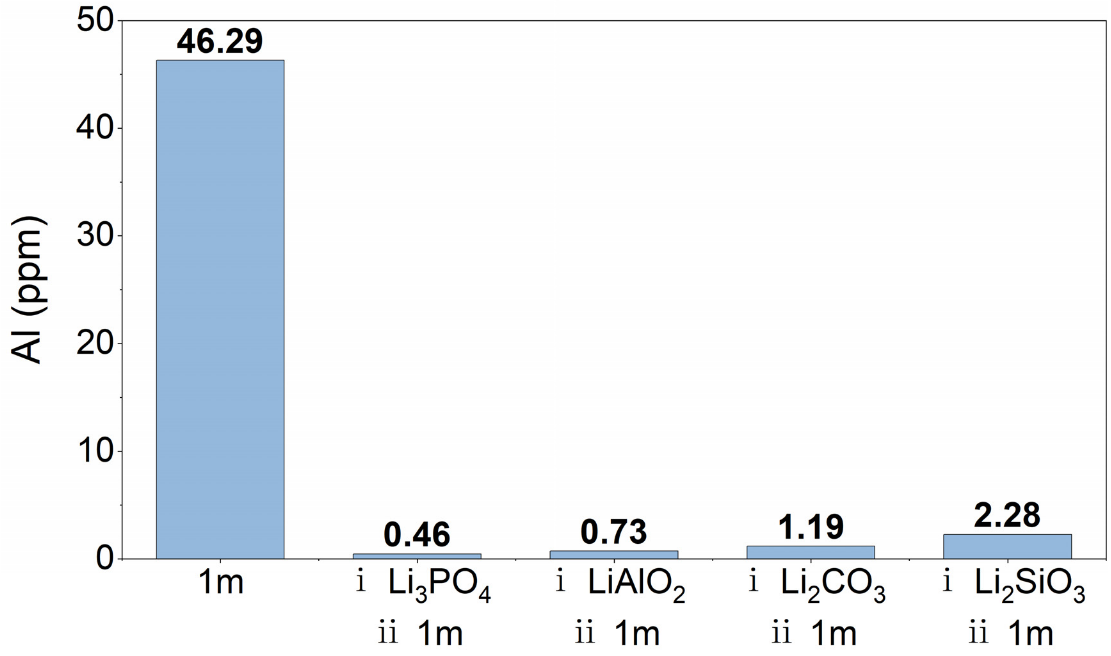

Figure S4. Al content in electrolytes by ICP measurement after CA experiments. i and ii represent the sequence of CA experiments conducted using different electrolytes. Eg: i Li_3PO_4 ii 1m means conducting the CA experiment with Al foil in 1m LiTFSI + Li_3PO_4 first, followed by conducting another CA experiment in 1m LiTFSI.

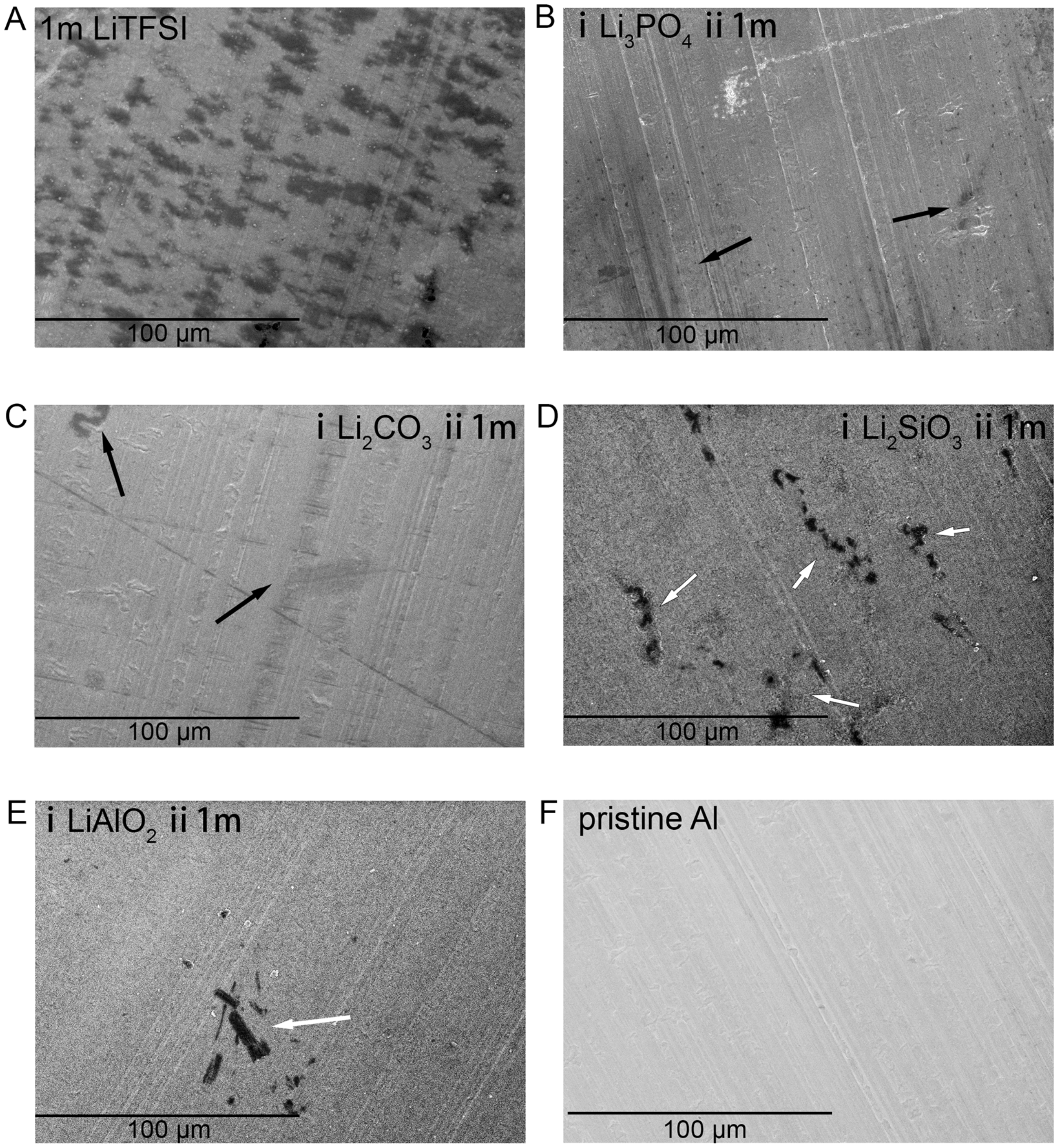

Figure S5. SEM images of Al foils after CA experiments. (A) Al foil after single CA experiment in 1m LiTFSI. (B)(C)(D)(E) Al foils that were conducted in CA experiments in 1m LiTFSI with saturated HTAs (Li_3PO_4, Li_2CO_3, Li_2SiO_3, and $LiAlO_2$) respectively first, and then conducted the same CA experiments in 1m LiTFSI. (F) pristine Al foil. i and ii represent the sequence of CA experiments conducted using different electrolytes. Eg: i Li_3PO_4 ii 1m means conducting the CA

experiment with Al foil in 1m LiTFSI + Li_3PO_4 first, followed by conducting another CA experiment in 1m LiTFSI.

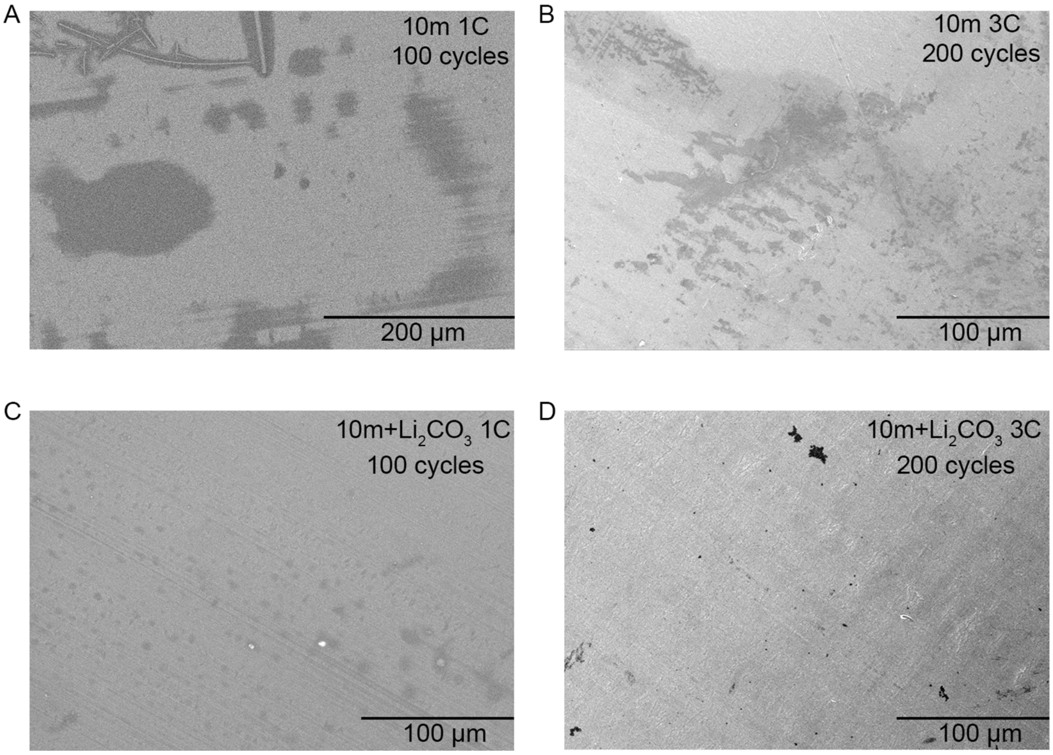

Figure S6. SEM photos of Al current collector of cathode after $LiMn_2O_4$-TiO_2 batteries cycling. (A) 10m LiTFSI, battery underwent 100 cycles at 1C. (B) 10m LiTFSI, battery underwent 200 cycles at 3C. (C) 10m LiTFSI with Li_2CO_3, battery underwent 100 cycles at 1C. (D) 10m LiTFSI with Li_2CO_3, battery underwent 200 cycles at 3C.

Repeatability of the CA experiments

The corrosion experiments in Figure 2 were repeated two more times, and we compiled some of the data to create a line graph with error bars as shown in Figure S7 and Figure S8. The repeatability of the corrosion experiments is good, and it closely matches the raw data in Figure 2. After adding HTA, there is a significant reduction in corrosion current density, and both Li_2CO_3 and Li_3PO_4 consistently perform well. To make the data of CA experiments earlier to process, we choose the average corrosion current density of the last section of the 4.5V vs Li/Li^+ stage of CA experiments to represent its corrosion extent in electrolytes, as shown in Figure S7 and S8.

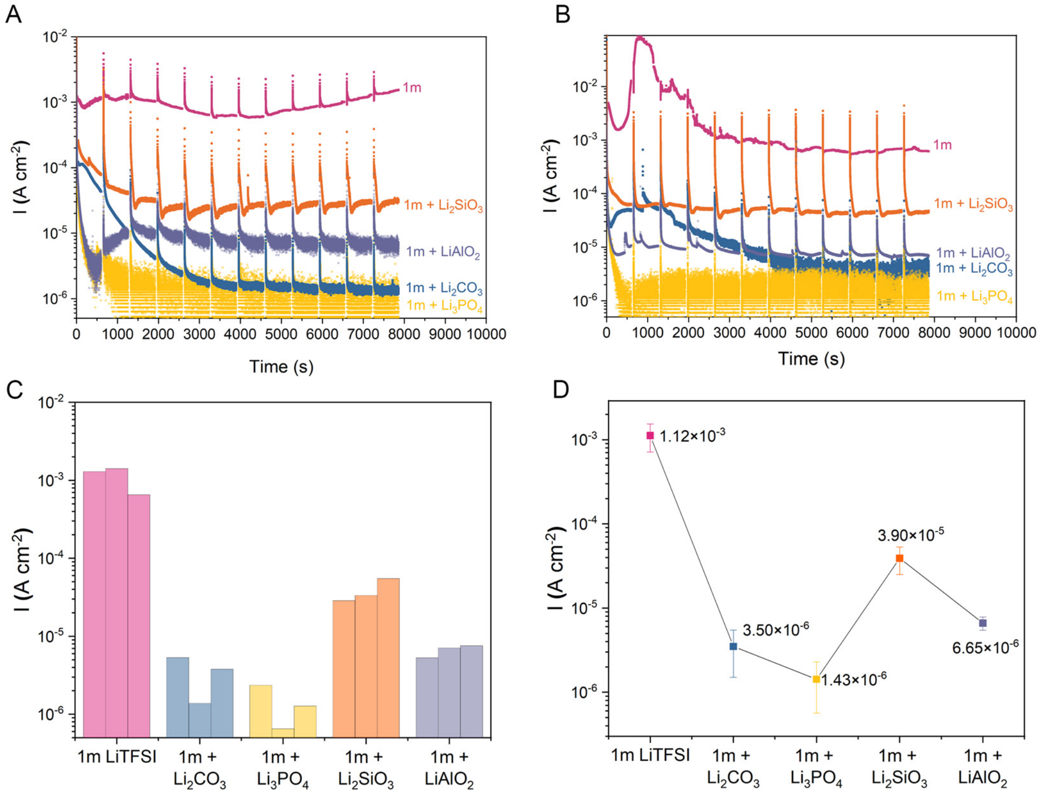

Figure S7. The repeatability of the corrosion chronoamperometry experiments of Al in 1m LiTFSI solutions with additives. (A)(B) Two sets of repeated experiments as in Figure 2A. (C) The average current density of the last segment of the CA experiment in each of the three trials. (D) Statistical averaging and error analysis of the data in (C). Error bar in (D) are obtained by calculating the mean and adding/subtracting the standard deviation from the three data in (C).

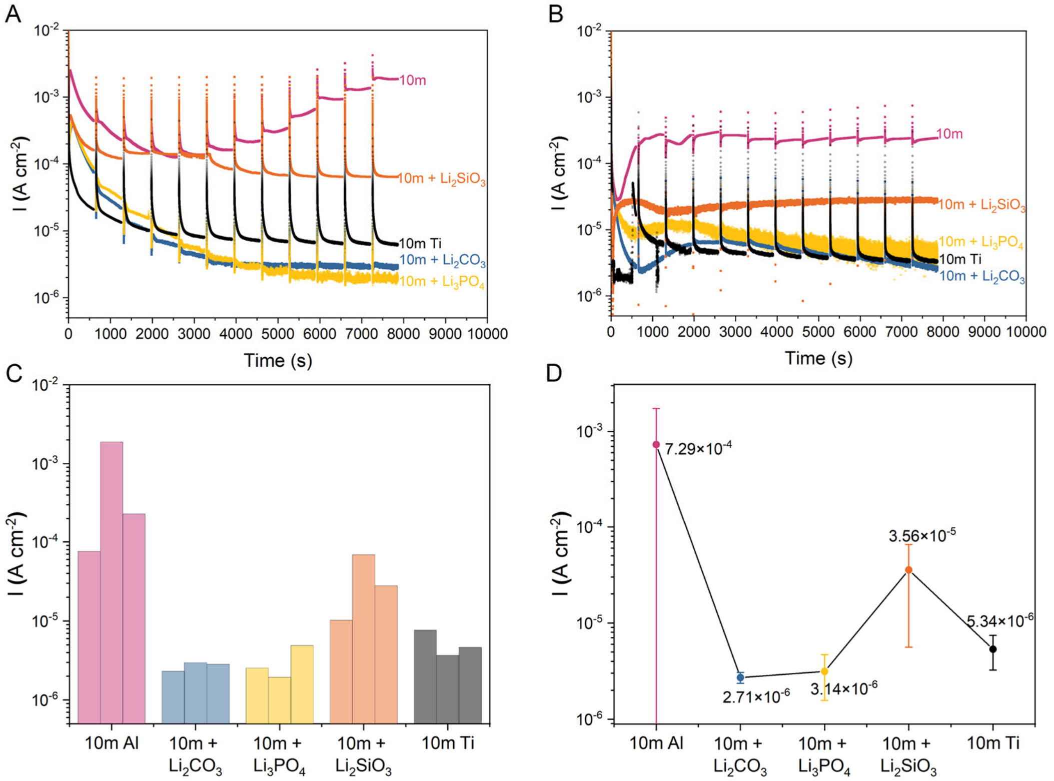

Figure S8. The repeatability of the corrosion chronoamperometry experiments of Al in 10m LiTFSI solutions with additives. (A)(B) Two sets of repeated experiments as in Figure 2B. (C) The average current density of the last segment of the CA experiment in each of the three trials. (D) Statistical averaging and error analysis of the data in (C). Error bar in (D) are obtained by calculating the mean and adding/subtracting the standard deviation from the three data in (C).

The verification of the anti-corrosion effect of Na_2CO_3 and Li_2CO_3 in 1m NaTFSI

The effect of anions is comprehensively discussed in the test. To check the influence of cation on the anti-corrosion effect, we conducted CA experiments in 1m NaTFSI solution at 4.5V vs Li/Li^+ lasting 14h, and the electrode potential was relaxed to open the circuit for 1 minute every 10 minutes at 4.5V. Saturated Li_2CO_3 and 0.05m Na_2CO_3 were added, respectively, as additives. As shown in Figure S9, electrolytes with Na_2CO_3 passivated Al as the test proceeded, and the corrosion current density gradually reduced, while Al in pure NaTFSI solution corroded gradually and the current density kept rising. Al content in electrolyte decreases significantly with Na_2CO_3, and Al foil is intact with few pitting corrosion marks, while Al foil was corroded a hole in pure 1m NaTFSI solution in Figure S11. It can be inferred that CO_3^{2-} can still protect Al after changing the cation in the electrolyte. On the other hand, Al was kept stable in the electrolyte with Li_2CO_3 all the time, with a very low corrosion current density and the lowest Al content in the electrolyte. The anti-corrosion effect of Li_2CO_3 is better than that of Na_2CO_3. The reason is unclear. More research should be done in the future.

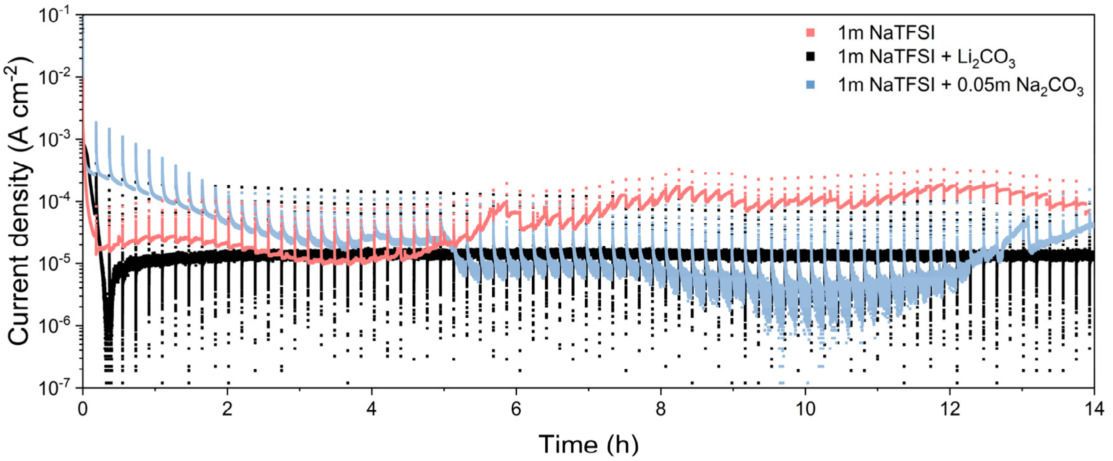

Figure S9. The verification of the anti-corrosion effect of Na_2CO_3 and Li_2CO_3 in 1m NaTFSI. CA experiments were carried out at 4.5V vs Li/Li$^+$ on Al current collectors for 14h in 1m NaTFSI , 1m NaTFSI + 0.05m Na_2CO_3 and 1m NaTFSI + saturated Li_2CO_3.

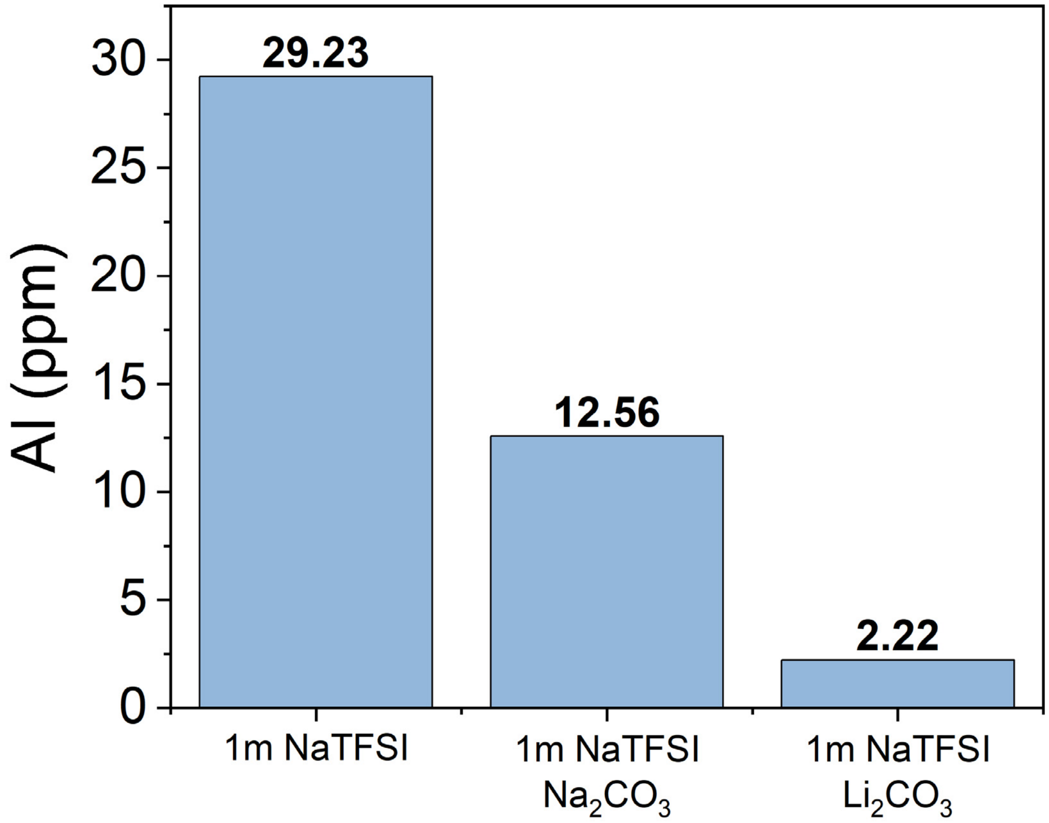

Figure S10. Al content in electrolyte after CA experiments. CA experiments were carried out at 4.5V vs Li/Li$^+$ on Al current collectors for 14h in 1m NaTFSI, 1m NaTFSI + 0.05m Na_2CO_3 and 1m NaTFSI + saturated Li_2CO_3.

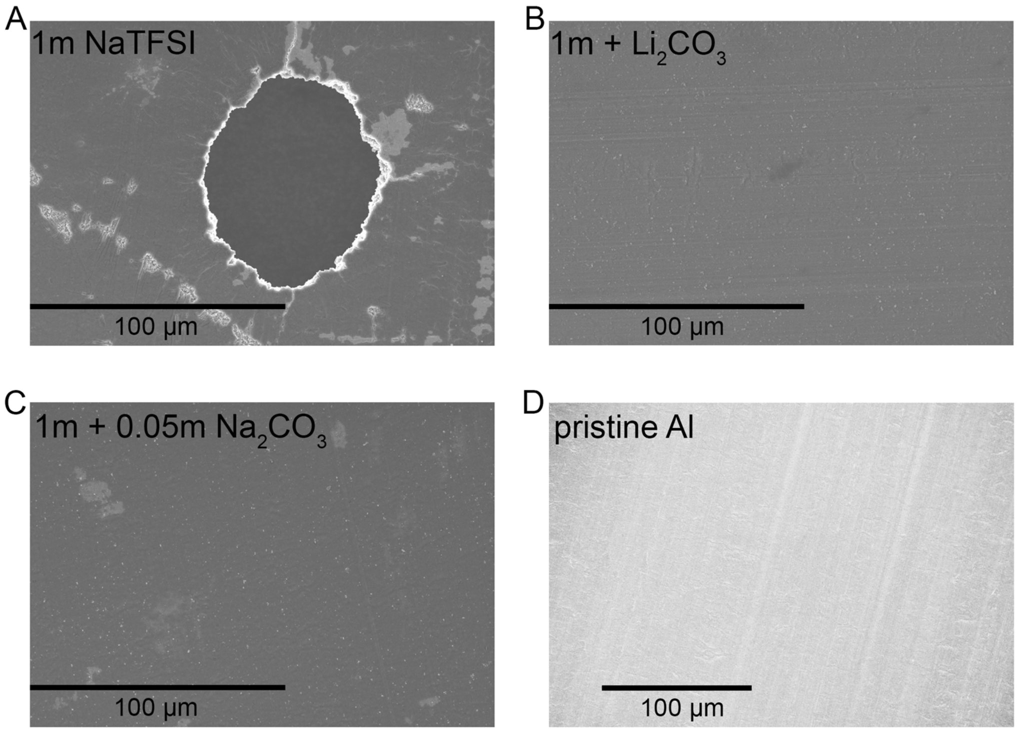

Figure S11. SEM images of Al foils after CA experiments. (A) Al foil in 1m NaTFSI. (B) Al foil in 1m NaTFSI with saturated Li_2CO_3. (C) Al foil in 1m NaTFSI with 0.05m Li_2CO_3. (D) pristine Al foil.

Supplementary Note 3. Identify and quantify the Al oxidation corrosion for lithium compensation

Identify the Al corrosion in the battery curves.

The potentials of Al oxidation and Li extraction from over-lithiated $LiMn_2O_4$ are close but distinguishable. To avoid all possibilities of confusion, we designed the experiments in Figure 4 that charge $LiMn_2O_4$-TiO_2-TiO_2 cells twice in a row without discharging to demonstrate that passivation almost happens at the initial cycle. Here is the detailed analysis of the potential difference between Al oxidation and Li extraction from over-lithiated $LiMn_2O_4$.

The corrosion of Al current collector almost only happens in the first cycle. It keeps stable after passivation by an anti-corrosion additive. From the second cycle, the first charging plateau at around 1.2V is almost the lithium extraction from the $LiMn_2O_4$ over-lithiation, lasting many cycles until the over-lithiation in $LiMn_2O_4$ is consumed up by the side reactions at anode-like hydrogen evolution reaction (HER). There are remarkable differences between the first and 10th charging curves in Figure S12. Δ means the difference between the potential of Al corrosion in the first cycle and the potential of lithium extraction in the 10th cycle from the over-lithiated $LiMn_2O_4$. Δ is about 0.12V with Li_2CO_3 and 0.13V with Li_3PO_4, which is clear enough to distinguish Al corrosion and $LiMn_2O_4$ over-lithiation (Figure S12), and the curve of the latter is very flat, while the Al corrosion curve is quite oblique, which means passivation happens at the same time to make oxidation harder. Without the help of additives, Al in 10m LiTFSI is hard to passivated. That is why its voltage difference is 0.05V, less than that with additive. Though it is distinguishable, it is better to simplify our demonstration. We design a consecutive charge process in Figure 4 to avoid confusion from over-lithiated $LiMn_2O_4$.

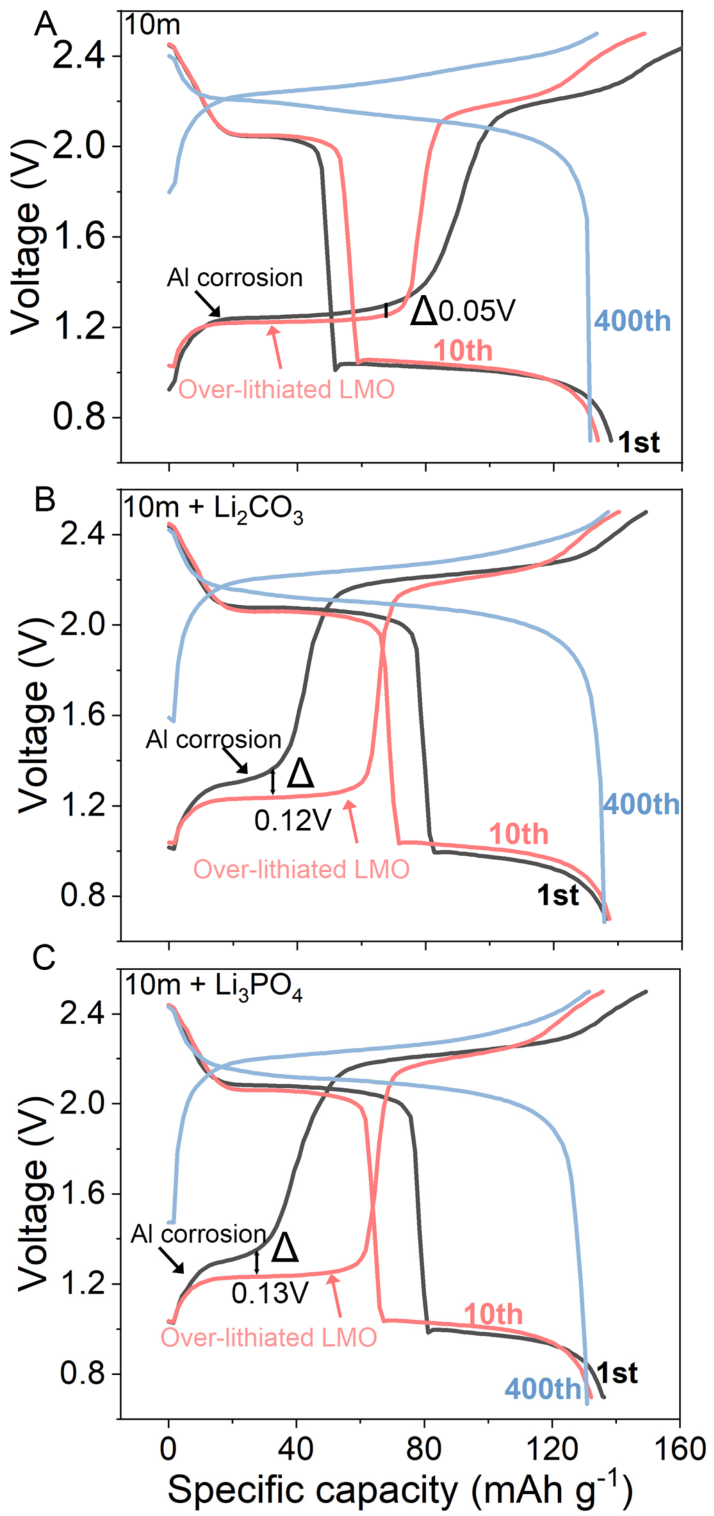

Figure S12. The potential difference between Al passivation and Li extraction from over-lithiated $LiMn_2O_4$. (A) 10m LiTFSI as electrolyte. (B) (C) 10m LiTFSI with Li_2CO_3 or Li_3PO_4 as electrolyte. Rate is 3C.

Quantify the Al corrosion for lithium compensation

In the text, we attach an extra bare 2cm^2 Al foil behind the $LiMn_2O_4$ electrode to magnify the lithium supplement phenomenon and clarify the mechanism when using Al current collector at the cathode in Figures 3, 4, 5. The extra bare Al foil is bigger than the $LiMn_2O_4$ electrode, which prolongs the battery life. Because of side reactions, it's hard to get accurate capacity from Al oxidation. To determine how much capacity Al could provide, we constructed pouch cells with 2cm^2 bare Al foil as the cathode and TiO_2 as the anode. Charge the pouch cell at a constant 2.4V voltage for 20 minutes, then discharge at 1C to check if TiO_2 gets intercalated and cycle it 10 times. This experiment clarifies that the passivation of Al can get TiO_2 intercalated at 2.4V in Figure S13 ABC because the cells have considerable capacity at the discharging process. Taking the charging capacity to quantize Al oxidation in Figure S13 is reasonable. To make a comparison, the charging capacity of the first three cycles is shown in Figure S14A. The capacity from Al oxidation in Figure S14A meets the expectation that it keeps getting lower in the following cycles because electrolyte with additive helps passivate Al.

Batteries with Al current collector from the section above also contain an extra 2cm^2 bare Al foil, so we take the maximum over-lithiation capacity of $LiMn_2O_4$ during normal cycling to represent capacity from Al passivation in Figure S14B to make a comparison. Though the data is underestimated owing to side reactions like HER and the formation of SEI, it is closely related to the capacity from Al oxidation. The lithium supplement in practical in Figure S14B is consistent with that in Figure S14A. It can be inferred that 2cm^2 bare Al in 10m LiTFSI with anti-corrosion additive could provide about 0.1 mAh capacity at the potential of $LiMn_2O_4$ for 20 minutes. However, many double-coated Al current collectors are stacked in practical industrial production. The data in the section above is incomprehensive to assess the effect of lithium supplement by real Al current collector in the commercial battery. In actual battery production, Al foil is coated by active material on both sides, which reduces its contact with electrolytes and weakens the lithium supplement effect. To get a more objective

conclusion, we assembled three pouch cells with $LiMn_2O_4$ coated on both sides of $2cm^2$ Al foil as cathode and took the maximum over-lithiation capacity of $LiMn_2O_4$ as a reference. The capacity from Al gets much lower in comparison with that in Figures S13A and S13B. After getting rid of the loss of side reactions like HER and SEI formation, passivation of the $2cm^2$ Al current collector at the charging process that $LiMn_2O_4$ coats on both sides can get $LiMn_2O_4$ over-lithiated for about 0.05 mAh in 10m LiTFSI with additive. Al current collector's capacity is insufficient to support our ideal long-life-time ALIB for industrial application. That is why we propose a new battery design with an extra sacrificial Al electrode in Figure 6. The new design separates the pre-lithiation and normal charge processes, which means a harsher environment could be applied to the sacrificial Al electrode at the pre-lithiation process without worrying about the influence of traditional cathode and anode, such as high voltage and temperature.

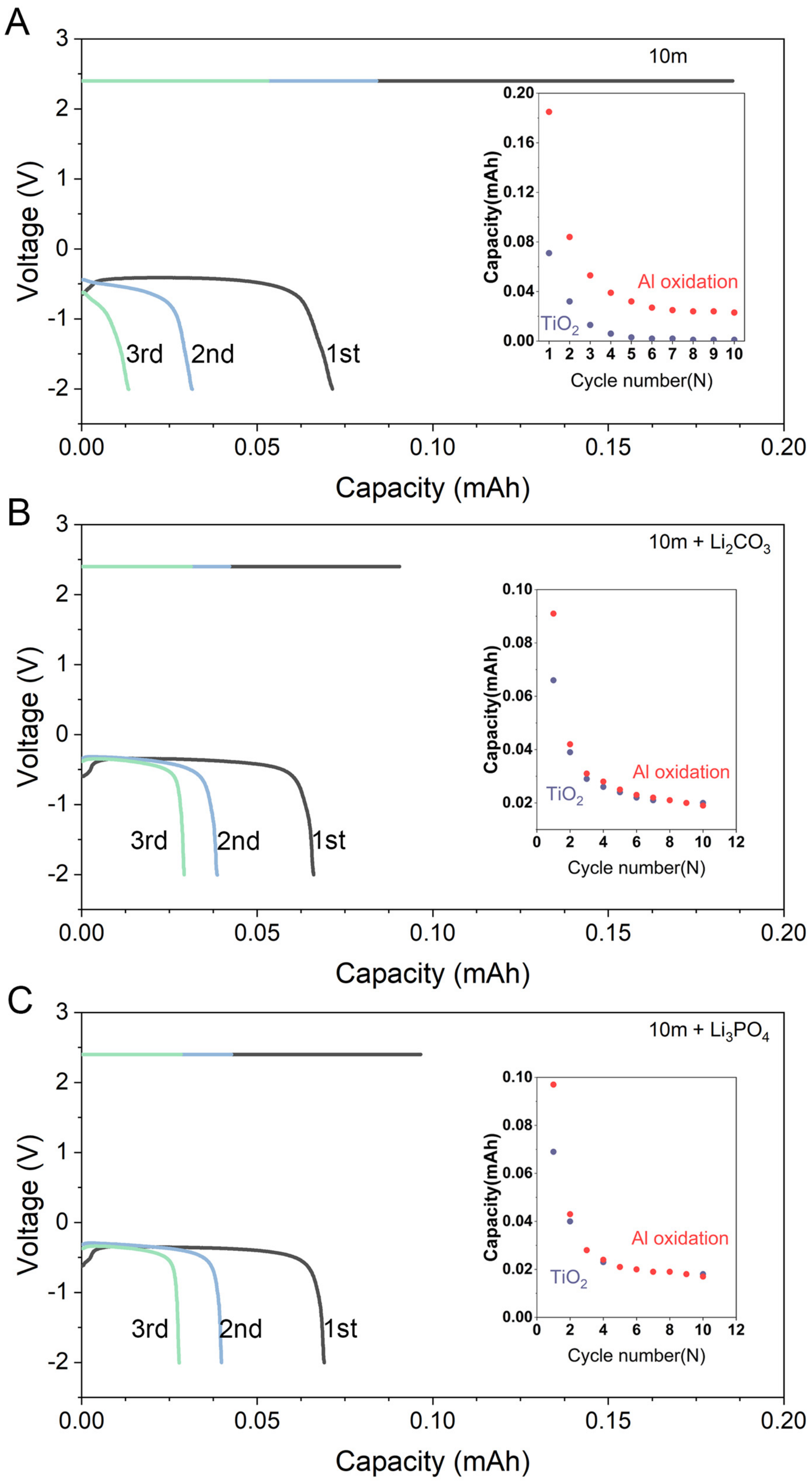

Figure S13. The capacity of Al oxidation in Al-TiO_2 pouch cell. (A)(B)(C) 2.4V constant voltage charging for 20 minutes and 1C constant current discharging profiles of the first three cycles with electrolyte 10m LiTFSI, 10m LiTFSI + Li_2CO_3, or 10m LiTFSI + Li_3PO_4, respectively, with the capacity-cycle number plot inside. The area of Al is 2cm^2. The inset graph corresponds to the cyclic

performance of the Al-TiO_2 battery for 10 cycles (2.4V constant voltage charge, 1C discharge).

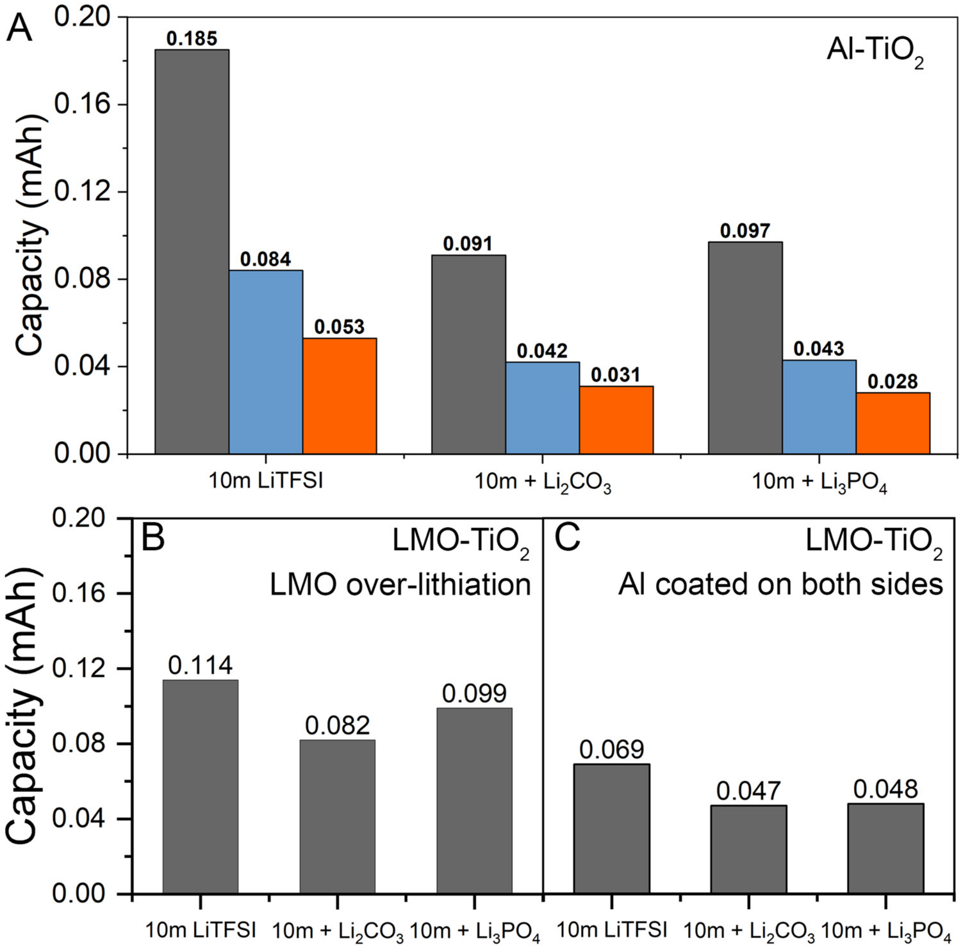

Figure S14. Oxidation capacity of 2cm² Al in different conditions. (A) Oxidation capacity of Al in the first three cycles of Al-TiO_2 battery at 2.4V constant voltage for 20 minutes. (B) The over-lithiation capacity of LMO with 2cm² extra bare Al foil attached behind the LMO electrode. (C) The over-lithiation capacity of LMO coated on both sides of the 2cm² Al current collector.

Supplementary Note 4. Illustrate the prototype of self-prolonging aqueous Li-ion batteries and check the performance of Al current collector in the cycling.

Validating the feasibility of the sacrificial pre-lithiation Al electrode in the battery

It is hard for double-coated Al current collector with a high loading mass to provide enough capacity with an anti-corrosion additive as indicated in Figure S14. The condition of the normal charge process is mild for Al current collector protected by an anti-corrosion additive. To make the pre-lithiation idea more practical in industrial applications, we design a new type of battery with an extra sacrificial Al electrode in Figure S15. Benefiting from this design, the normal charging process and the pre-lithiation process can be separated with different conditions. To verify this design, we constructed a small pouch cell with Ti current collector coated $LiMn_2O_4$ as cathode and an extra sacrificial Al electrode for pre-lithiation. The choice of Ti current collector is to verify the role of sacrificing Al electrode, making the sacrificial Al electrode the sole Al source to be corroded, thereby ensuring that the pre-lithiation capacity of TiO_2 comes solely from the sacrificial Al electrode. Before battery cycling, TiO_2 is prelithiated by the sacrificial Al electrode at a constant 2.4V potential for 20mins. Since Ti is hard to be oxidized at the charging process, if the plateau of over-lithiated $LiMn_2O_4$ only appears at the discharging process at the initial cycle, it must be contributed by the pre-lithiated process before. As shown in Figure S15B, a distinct plateau of over-lithiated $LiMn_2O_4$ appears at the first discharge process, which verifies the feasibility of this design. The Al-TiO_2 cell in Figure S13 also demonstrated that the Al electrode could get TiO_2 lithiated. In practical applications with a high mass loading, sacrificial Al electrode can be sandpapered to make it more vulnerable and be applied by a high voltage at high temperature to speed up the pre-lithiation process and contribute more capacity.

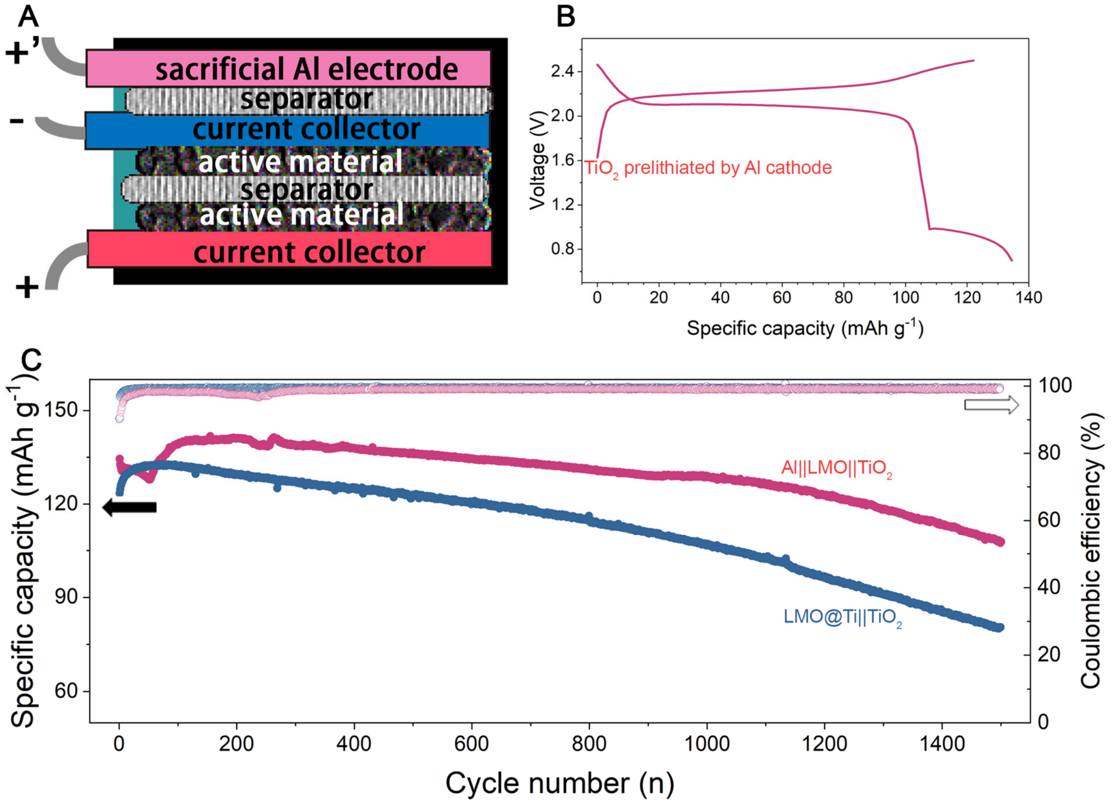

Figure S15. The prototype of self-prolonging aqueous Li-ion batteries (Al||$LiMn_2O_4$||TiO_2). (A) the schematic diagram of the self-prolonging ALIBs. (B) the first charge-discharge profiles of self-prolonging ALIBs after the pre-lithiation of TiO_2 anode at 2.4V voltage for 20mins by the sacrificial Al electrode. (C) the cycle stability of SP-ALIBs (Al||$LiMn_2O_4$||TiO_2) and traditional $LiMn_2O_4$||TiO_2 battery in 10m LiTFSI + Li_2CO_3. Both use Ti as a current collector for $LiMn_2O_4$ while Al is for TiO_2.

Repeatability verification of 0.5Ah SP-ALIB

To make the conclusions more reliable, the 0.5Ah batteries cycling at a rate of 0.1C is presented in Figure S16. We made a total of three 0.5Ah SP-ALIBs batteries to validate the reliability of the cycling stability at 1C in Figure S17, and the consistency was excellent.

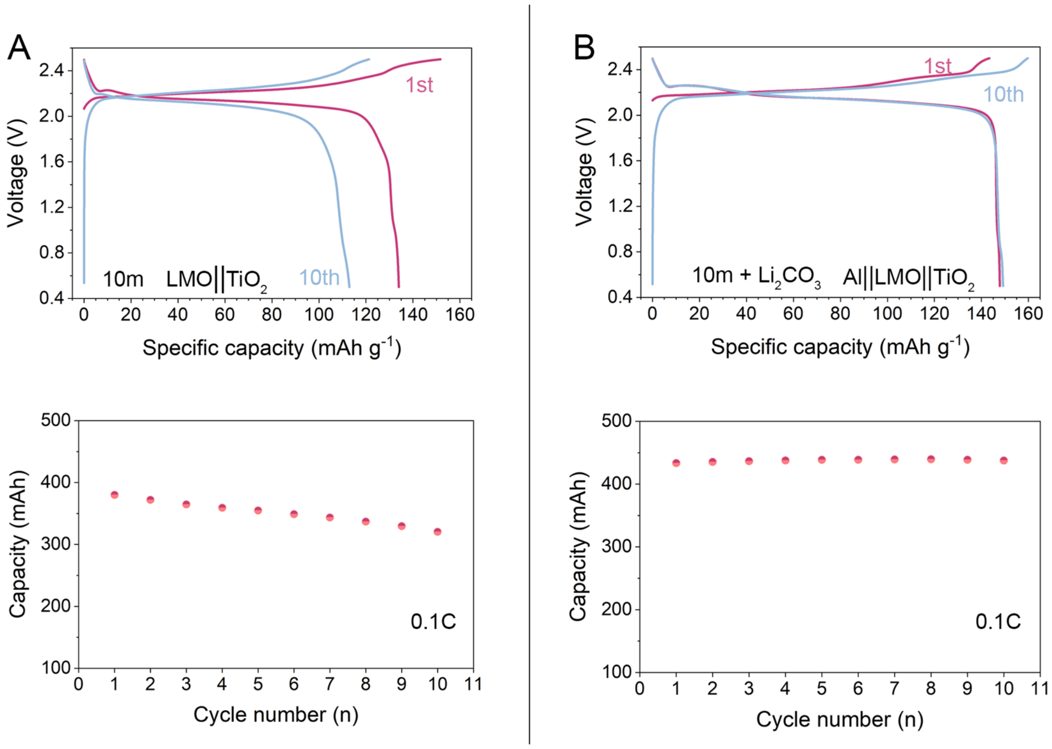

Figure S16. The cycle of the 0.5Ah batteries at 0.1C. (A) the charge-discharge profile and cycle performance of the normal $LiMn_2O_4$-TiO_2 cell with 10m LiTFSI electrolyte. (B) the charge-discharge profile and cycle performance of SP-ALIB (Al||LMO||TiO_2) with 10m LiTFSI + Li_2CO_3.

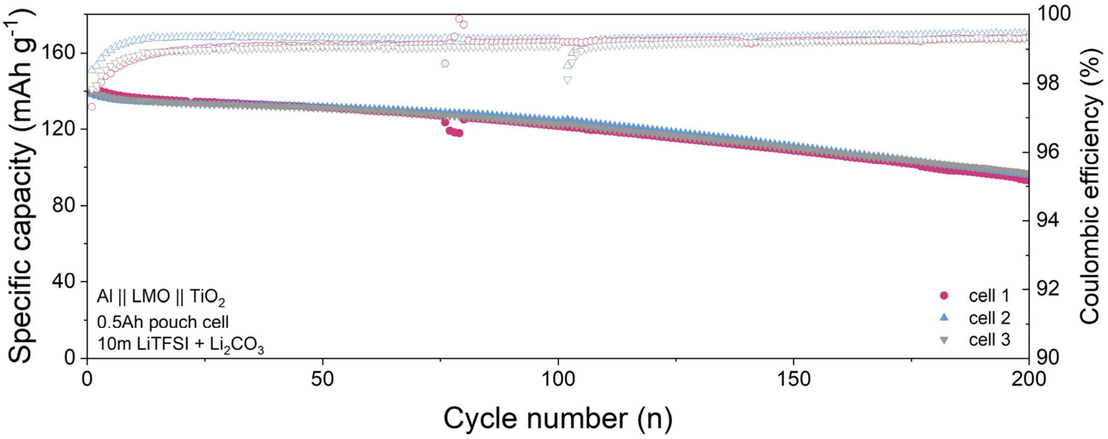

Figure S17. The repeatability of the 0.5Ah Al||LMO||TiO_2 pouch cells with 10m LiTFSI + Li_2CO_3 at 1C.

The over-lithiation plateau in 0.5 Ah $LiMn_2O_4$-TiO_2 pouch cell

We do not excessively get TiO_2 pre-lithiated in the formation process, so after offsetting the side reactions of SEI formation and hydrogen evolution, the excess capacity of over-lithiated $LiMn_2O_4$ is insignificant, as shown in Figure S18. Therefore, though the voltage plateau of the over-lithiated $LiMn_2O_4$ is low, it does not impact the battery's energy density much.

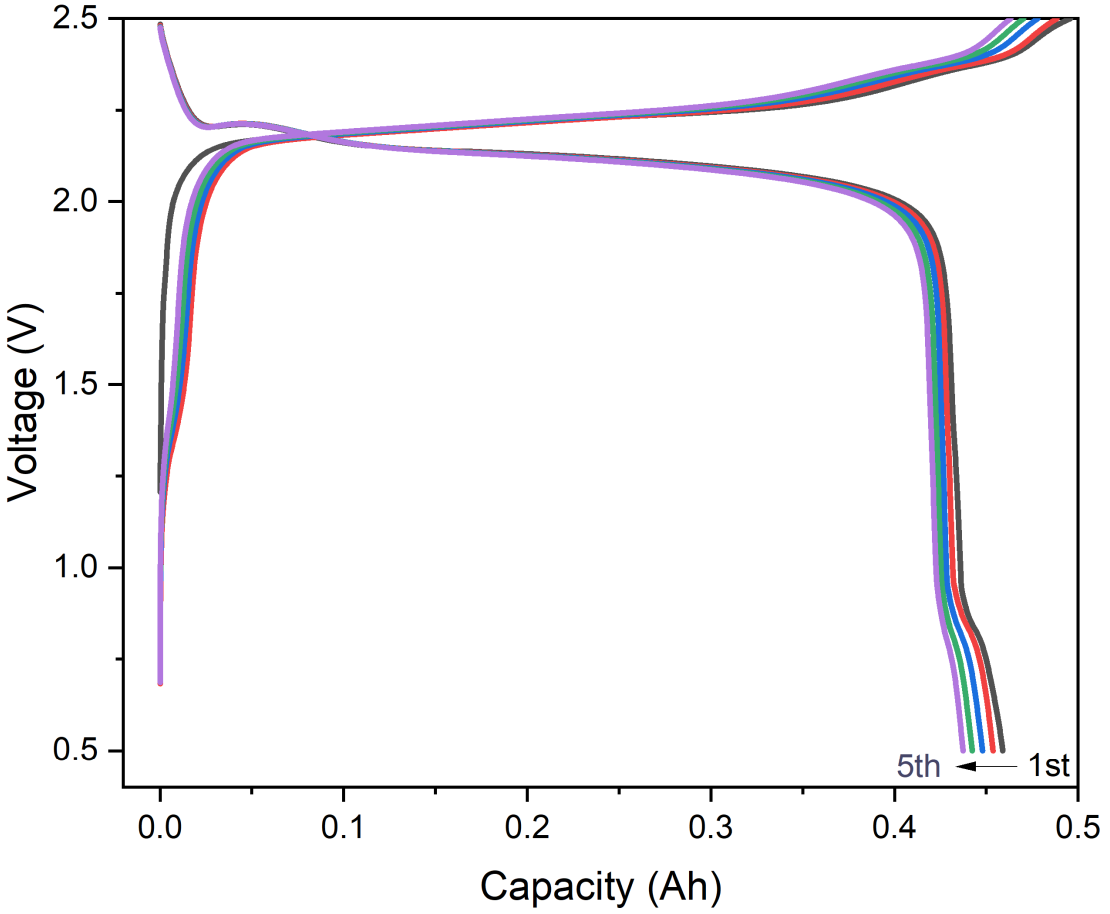

Figure S18. The over-lithiation plateau in 0.5 Ah $LiMn_2O_4$-TiO_2 pouch cell. This SP-ALIB battery is pre-lithiated by the sacrificial pre-lithiation Al electrode first, and then cycled. Electrolyte is 10m LiTFSI + Li_2CO_3.

The cycling stability comparison of Al + HTA with Ti current collector without over-lithiation.

Cycling stability comparison of Al current collector with Li_2CO_3 and Ti current collector is shown in Figure S19. There is no over-lithiation shown in the first cycle. The capacity compensation from the electrochemical passivation of the Al current collector is not sufficient to offset the capacity loss caused by SEI formation and hydrogen evolution side reactions because there is no additional Al foil or the third sacrificial Al electrode with specific formation process. Therefore, it may not be reflected in the curves (over-lithiation), but it seems to play some role.

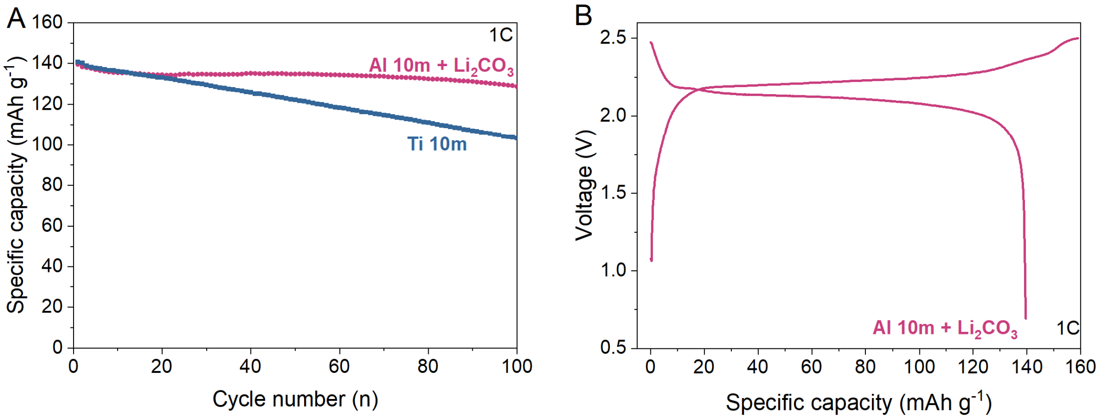

Figure S19. Small pouch cell cycling stability of Al current collector with 10m LiTFSI + Li_2CO_3 vs Ti current collector with 10m LiTFSI. (A) Cycling stability of $LiMn_2O_4$-TiO_2 batteries. (B) The charge-discharge profile of the first cycle for the battery with Al current collector using 10m + Li_2CO_3. No additional Al foil or the sacrificial Al electrode.

Reference:

1 Aluminum statistics and information. https://www.usgs.gov/centers/national-minerals-information-center/aluminum-statistics-and-information (2022).

2 North America stainless steel price forecasts. https://mepsinternational.com/gb/en/products/north-america-stainless-steel-price-forecasts (2022).

3 Nickel price (I:NPNM). https://ycharts.com/indicators/nickel_price (2022).

4 Titanium Statistics and information. https://www.usgs.gov/centers/national-minerals-information-center/titanium-statistics-and-information (2022).
