# Supplementary material for: Aluminum corrosion–passivation regulation prolongs aqueous batteries life
Source: Nat Commun. 2024 Apr 4;15:2922. doi: 10.1038/s41467-024-47145-3 (PMC10995134; doi:10.1038/s41467-024-47145-3)
Supplement: Supplementary file 3 — Reporting Summary [file 41467_2024_47145_MOESM3_ESM.pdf]

nature portfolio

Corresponding author(s): Liumin Suo

Last updated by author(s): Feb 25, 2024

Reporting Summary

Nature Portfolio wishes to improve the reproducibility of the work that we publish. This form provides structure for consistency and transparency in reporting. For further information on Nature Portfolio policies, see our Editorial Policies and the Editorial Policy Checklist.

Statistics

For all statistical analyses, confirm that the following items are present in the figure legend, table legend, main text, or Methods section.

n/a	Confirmed	
☐	☒	The exact sample size (*n*) for each experimental group/condition, given as a discrete number and unit of measurement
☐	☒	A statement on whether measurements were taken from distinct samples or whether the same sample was measured repeatedly
☒	☐	The statistical test(s) used AND whether they are one- or two-sided<br>*Only common tests should be described solely by name; describe more complex techniques in the Methods section.*
☒	☐	A description of all covariates tested
☒	☐	A description of any assumptions or corrections, such as tests of normality and adjustment for multiple comparisons
☐	☒	A full description of the statistical parameters including central tendency (e.g. means) or other basic estimates (e.g. regression coefficient) AND variation (e.g. standard deviation) or associated estimates of uncertainty (e.g. confidence intervals)
☒	☐	For null hypothesis testing, the test statistic (e.g. *F*, *t*, *r*) with confidence intervals, effect sizes, degrees of freedom and *P* value noted<br>*Give P values as exact values whenever suitable.*
☒	☐	For Bayesian analysis, information on the choice of priors and Markov chain Monte Carlo settings
☒	☐	For hierarchical and complex designs, identification of the appropriate level for tests and full reporting of outcomes
☒	☐	Estimates of effect sizes (e.g. Cohen's *d*, Pearson's *r*), indicating how they were calculated

Our web collection on statistics for biologists contains articles on many of the points above.

Software and code

Policy information about availability of computer code

Data collection	No software was used
Data analysis	No software was used

For manuscripts utilizing custom algorithms or software that are central to the research but not yet described in published literature, software must be made available to editors and reviewers. We strongly encourage code deposition in a community repository (e.g. GitHub). See the Nature Portfolio guidelines for submitting code & software for further information.

Data

Policy information about availability of data

All manuscripts must include a data availability statement. This statement should provide the following information, where applicable:

- Accession codes, unique identifiers, or web links for publicly available datasets
- A description of any restrictions on data availability
- For clinical datasets or third party data, please ensure that the statement adheres to our policy

All relevant data that support the findings of this study are presented in the article and Supplementary Information. Source data are available from the corresponding authors upon reasonable request.

Research involving human participants, their data, or biological material

Policy information about studies with human participants or human data. See also policy information about sex, gender (identity/presentation), and sexual orientation and race, ethnicity and racism.

Reporting on sex and gender	There is no human research participants in this work.
Reporting on race, ethnicity, or other socially relevant groupings	There is no human research participants in this work.
Population characteristics	There is no human research participants in this work.
Recruitment	There is no human research participants in this work.
Ethics oversight	There is no human research participants in this work.

Note that full information on the approval of the study protocol must also be provided in the manuscript.

Field-specific reporting

Please select the one below that is the best fit for your research. If you are not sure, read the appropriate sections before making your selection.

☐ Life sciences ☐ Behavioural & social sciences ☒ Ecological, evolutionary & environmental sciences

For a reference copy of the document with all sections, see nature.com/documents/nr-reporting-summary-flat.pdf

Ecological, evolutionary & environmental sciences study design

All studies must disclose on these points even when the disclosure is negative.

Study description	The aluminum current collector is widely used in non-aqueous batteries thanks to its cost-effectiveness, lightweight, and ease of fabrication. However, it is excluded from aqueous batteries due to its severe corrosion in the aqueous solution. Here, we come up with hydrolyzation-type anodic additives to form a robust passivation layer to suppress the corrosion, dramatically lowering the corrosion current density of aluminum by nearly three orders of magnitude to ~10-6 A cm-2. Besides, inspired by the discoveries that the electrochemical corrosion accompanied the anode pre-lithiation, we propose the prototype of self-prolonging aqueous Li-ion batteries (Al||LiMn2O4||TiO2) by additionally introducing a sacrificial aluminum electrode where the electrochemical corrosion is utilized as an electron supplement to prolong the cycling life of aqueous batteries, whose capacity retention rises from 49.5% to 70.1% after 200 cycles. Our work addresses the short-life issue of aqueous batteries resulting from current collector corrosion and lithium loss from side reactions.
Research sample	The Al foils used in the experiments can be purchased from commercial sources, and the Al foil used for testing and assembling the battery is from the same batch of material. All Details were explained in the Results and discussion and Methods section of the manuscript.
Sampling strategy	N/A
Data collection	We used data that was collected using standard practice in this type of work, and we have given detailed descriptions as to how this collection is carried out, in Methods of the manuscript
Timing and spatial scale	The samples were collected at predetermined times, which have given in the main text and Methods of the manuscript.
Data exclusions	N/A
Reproducibility	The findings in this paper were remarkably reproducible. The repetition of key data is presented in the Supplementary Information.
Randomization	N/A
Blinding	N/A

Did the study involve field work? ☐ Yes ☒ No

Reporting for specific materials, systems and methods

We require information from authors about some types of materials, experimental systems and methods used in many studies. Here, indicate whether each material, system or method listed is relevant to your study. If you are not sure if a list item applies to your research, read the appropriate section before selecting a response.

Materials & experimental systems

n/a	Involved in the study
☒	☐ Antibodies
☒	☐ Eukaryotic cell lines
☒	☐ Palaeontology and archaeology
☒	☐ Animals and other organisms
☒	☐ Clinical data
☒	☐ Dual use research of concern
☒	☐ Plants

Methods

n/a	Involved in the study
☒	☐ ChIP-seq
☒	☐ Flow cytometry
☒	☐ MRI-based neuroimaging

Plants

Seed stocks	*Report on the source of all seed stocks or other plant material used. If applicable, state the seed stock centre and catalogue number. If plant specimens were collected from the field, describe the collection location, date and sampling procedures.*
Novel plant genotypes	*Describe the methods by which all novel plant genotypes were produced. This includes those generated by transgenic approaches, gene editing, chemical/radiation-based mutagenesis and hybridization. For transgenic lines, describe the transformation method, the number of independent lines analyzed and the generation upon which experiments were performed. For gene-edited lines, describe the editor used, the endogenous sequence targeted for editing, the targeting guide RNA sequence (if applicable) and how the editor was applied.*
Authentication	*Describe any authentication procedures for each seed stock used or novel genotype generated. Describe any experiments used to assess the effect of a mutation and, where applicable, how potential secondary effects (e.g. second site T-DNA insertions, mosiacism, off-target gene editing) were examined.*
